# Percutaneous transhepatic duodenal drainage is good option for afferent loop syndrome for obstructive colorectal cancer patient with history of Billroth's operation II: A case report of a rare postoperative complication

**DOI:** 10.1002/ccr3.7725

**Published:** 2023-07-20

**Authors:** Tung‐Yuan Chen, Chin‐Wen Hsu, Yee‐Phoung Chang, Min‐Tsung Wang, Yueh‐Jung Wu, Ching‐Hsien Wang, Kuan‐Yu Wang, Tian‐Huei Chu, Yung‐Kuo Lee

**Affiliations:** ^1^ Division of Colorectal Surgery, Department of Surgery Kaohsiung Armed Forces General Hospital Kaohsiung Taiwan; ^2^ Division of General Surgery, Department of Surgery Kaohsiung Armed Forces General Hospital Kaohsiung City Taiwan; ^3^ Department of Cardiovascular Surgery Kaohsiung Armed Forces General Hospital Kaohsiung City Taiwan; ^4^ Department of Radiology Kaohsiung Armed Forces General Hospital Kaohsiung City Taiwan; ^5^ Medical Laboratory, Medical Education and Research Center, Kaohsiung Armed Forces General Hospital Kaohsiung City Taiwan; ^6^ Institute of Medical Science and Technology, National Sun Yat‐sen University Kaohsiung Taiwan

**Keywords:** afferent loop syndrome, colorectal cancer, colostomy, percutaneous transhepatic duodenum drainage

## Abstract

**Key Clinical Message:**

Temporal percutaneous transhepatic duodenum drainage (PTDD) seems to be effective in the treatment of postoperative afferent loop syndrome (ALS) following transverse loop colostomy for obstructive colorectal cancer.

**Abstract:**

Management of obstructive colorectal cancer still remains a challenge. There are various options with different risks of mortality and mobility for obstructive colorectal cancer. A rare unexpected postoperative ALS following a low anterior resection and transverse loop colostomy for obstructive colorectal cancer is presented in this report. A 64‐year‐old man had the acute ALS had been noted 10 days after transverse loop colostomy. An option was temporal PTDD treatment in the patient with history of Billroth's operation II for upper gastrointestinal bleeding 30 years ago. Acute ALS was treated by temporal PTDD. The drainage tube for PTDD was not removed until closure of the transverse colostomy 2 months later. The patient recovered uneventfully. Acute ALS after transverse loop colostomy for obstructive colorectal cancer is rare and has never been reported in the literature. The mechanism of acute ALS after construction of a loop colostomy and the treatment strategy of PTDD for acute ALS is presented.

## 1 INTRODUCTION

Obstructive rectal cancer is an emergency condition. The management still remains a challenge. The risks of ischemia bowel and perforation of colon may occur if the operation was delayed. There are various options with different risks of mortality and mobility for obstructive rectal cancer, such as stenting, one stage resection anastomosis, total colectomy, resection of obstructive rectal cancer plus a prophylactic loop colostomy, Hartmann's operation or simple colostomy.^1^, ^2^

Afferent loop syndrome (ALS) is usually developed as a complication after gastrectomy with gastrojejunostomy.^3^ But the acute ALS was found 10 days after T‐loop colostomy on our patient who experienced the B‐II operation 30 years ago. It was caused by traction of transverse loop colostomy and treated with temporal percutaneous transhepatic duodenum drainage (PTDD). The non‐surgical palliative PTDD is good option for acute afferent syndrome in status of T‐loop colostomy.

## 2 CASE PRESENTATION

**FIGURE 1:**
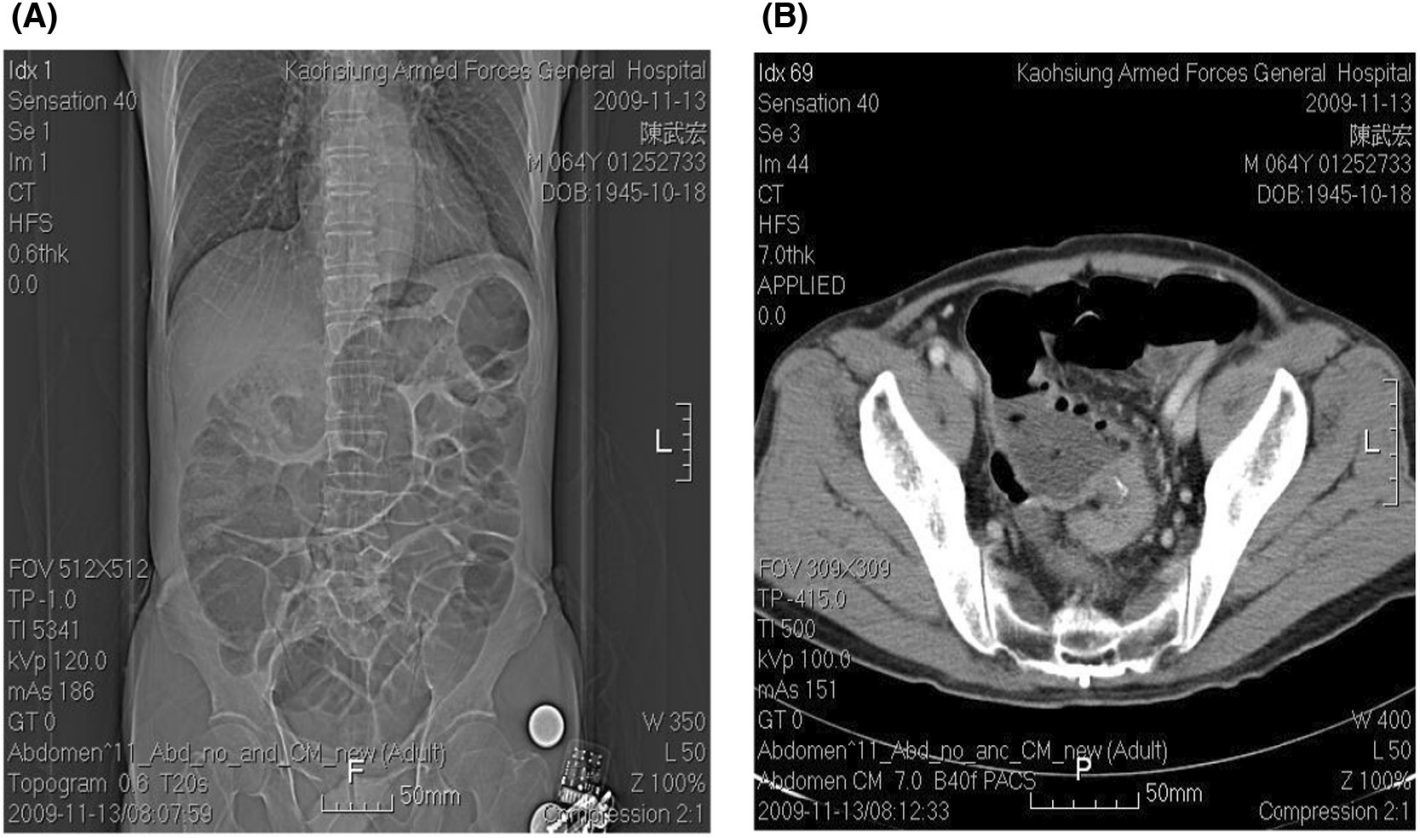
(A) Maximal dilation of colon without rectal air. (B) Rectal tumor with total obstruction.

**FIGURE 2:**
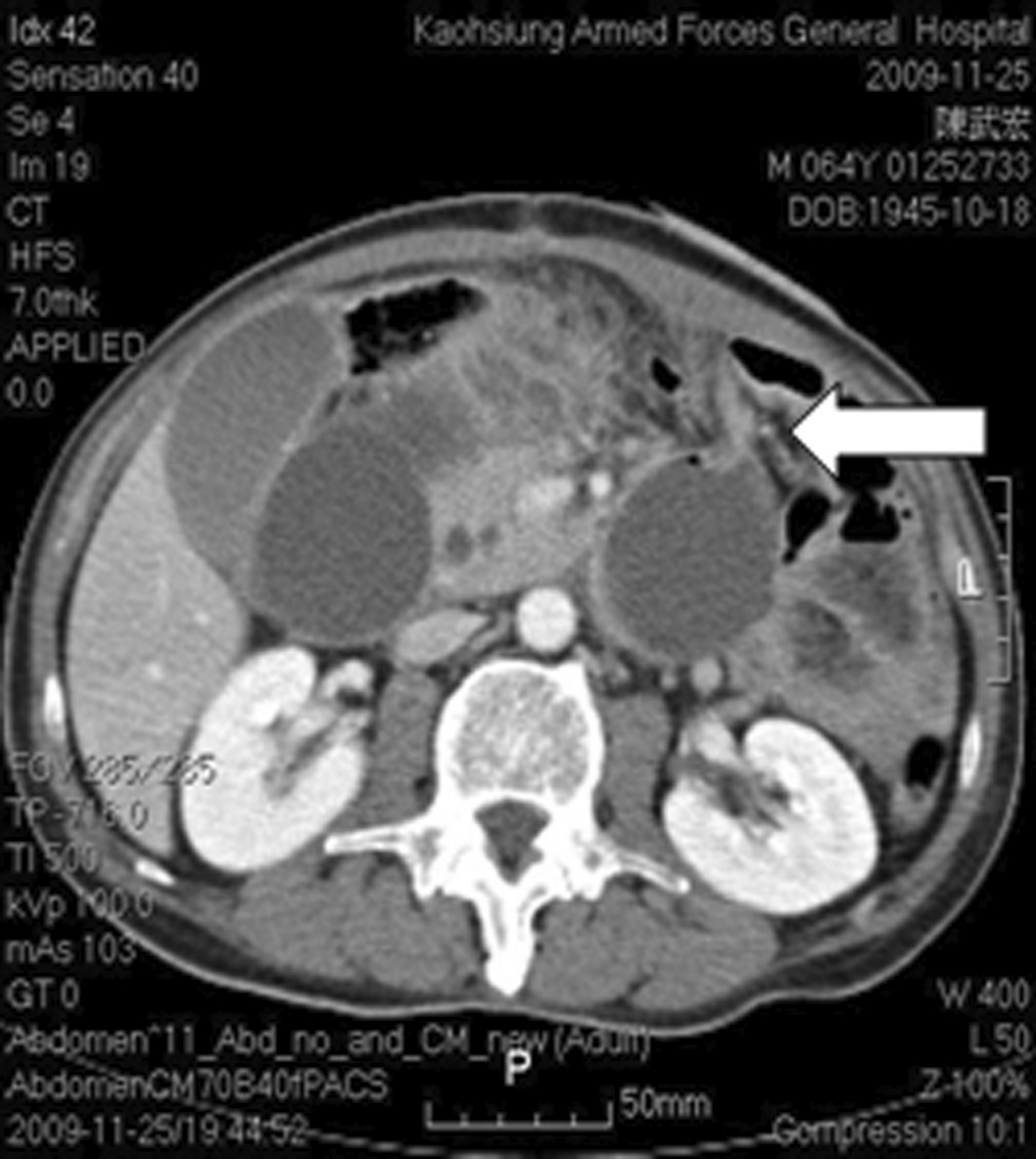
Obstructive lesion over 4th portion of duodenum near Treitz lig. White arrow shows the location of the obstruction.

**FIGURE 3:**
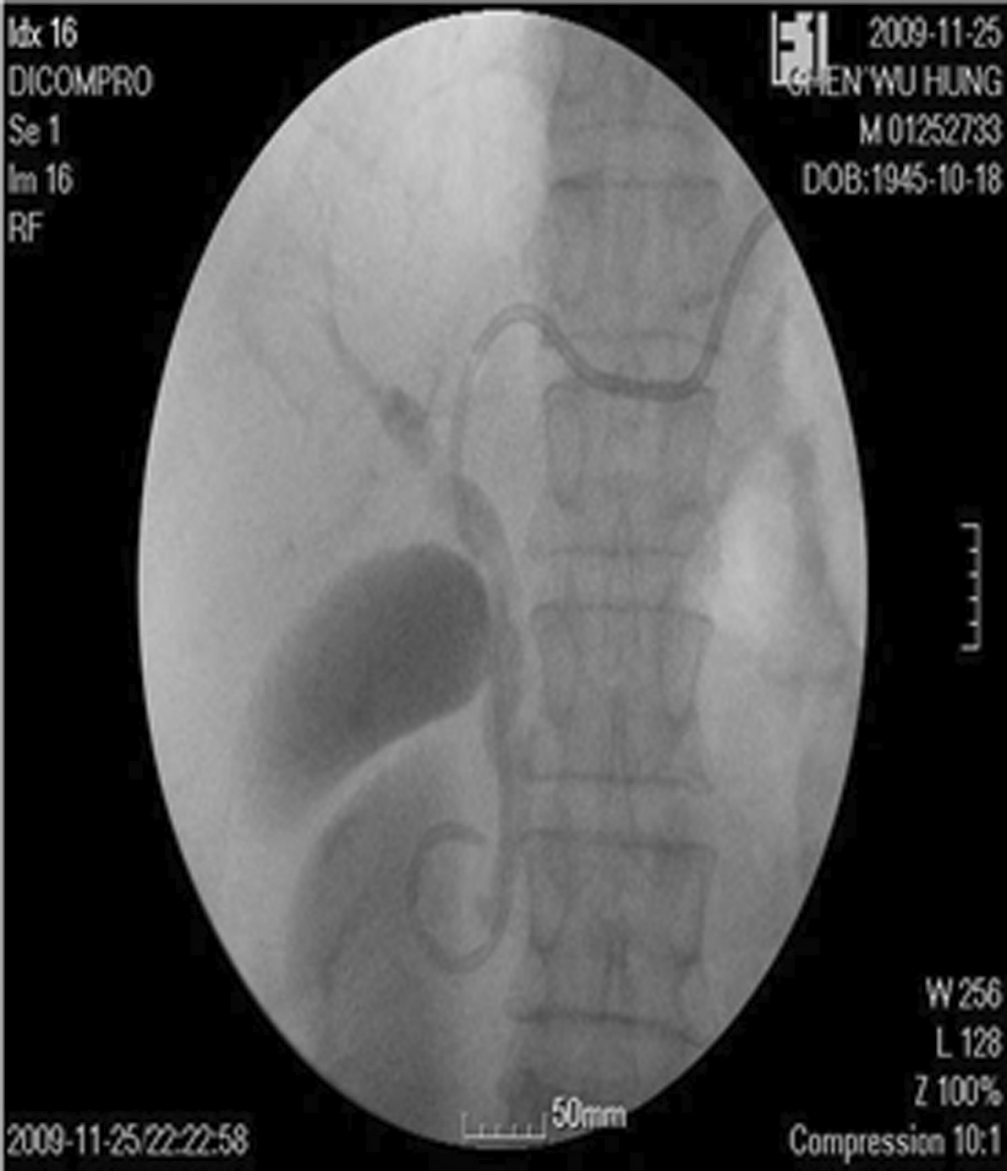
Percutaneous Transhepatic Duodenum Drainage (PTDD). Under sono‐guided and fluoro‐guided technique, a drainage tube (8‐Fr) was inserted pass through ampulla vater into the 2nd portion of duodenum via left intrahepatic bile duct (S3). The amount of drainage was 700 cc and 10 cc of the bile was cultured as usual.

A 64‐year‐old man was sent to our emergency room due to abdominal pain, fullness of abdomen and no stool passage for 4 days. He had history of peptic ulcer with bleeding post B‐II operation 30 years ago. The heart rate 94 beats/min, blood pressure 147/100 mmHg, and respiratory rate 20 breaths/min were found. Abdomen was tenderness without rebounding pain and ovoid in shape. An old surgical scar located upper mid‐line of abdomen. Metallic sound was noted. Kidney–ureter‐bladder abdominal radiography (KUB) and computed tomography (CT) of abdomen demonstrated intestinal obstruction with mass lesion over rectum‐sigmoid junction (Figure 1). Fiberoptic colonoscopy revealed an ulcerative solid mass around 4 cm in diameter causing totally obstruction which was 15 cm proximal to anus. Low anterior resection, on‐table lavage, anastomosis with EEA 29 (Ethicon; Johnson & Johnson), and prophylactic transverse loop colostomy had been performed right after colonoscopy. Antecolic anastomosis of previous gastrojejunostomy was noted while did the transverse loop colostomy. The colostomy was constructed 5 cm left to the umbilicus. Hiccup and fullness of abdomen were suffered at the 3rd day after surgery. Two shallow, active ulcers at LCS of remnant stomach were found near the anastomosis of gastrojejunostomy in panendoscopy at the 5th operation day. Afferent and efferent loops were noted as well. Liquid diet with 500–1000 mL/day was given in spite of persisting hiccup. Tachycardia with rate of 100–120 beats/min and abdominal pain over right upper quadrant (RUQ) region happened at the 9th operation day. Abrupt fever (temperature, 38.5°C) followed at the 11th day. The laboratory tests showed WBC was 12,100/mm3, amylase 214 U/L, and total bilirubin 5.1 mg/dL. Abdominal CT scan revealed afferent loop obstruction at the level of Treitz ligament with mild biliary tract dilation (Figure 2). The PTDD was performed right after percutaneous cholangial drainage (PTCD) (Figure 3). These symptoms were subsided successfully with PTDD and antibiotics. The amount of drainage from the drainage tube was about 1500–2000 mL/day and oral intake was well about 2000 mL/day. The laboratory data returned to normal range except amylase (about 200–400 U/L) and lipase (about 200–400 U/L). The patient was doing well with the PTDD tube for 5 weeks at home. The PTDD was removed while closure of transverse loop colostomy 7 weeks later. The patient was doing well till now for 5 years.

## 3 DISCUSSION

The cause of afferent loop obstruction can be benign or malignant lesions. Acute ALS usually accompanies with abrupt epigastric pain, non‐bilious vomiting in early phase of recovery. Chronic ALS usually occurs months or years after surgery, accompanying with partial obstruction and subsides with bilious vomiting 1–2 h after meal.^3^ ALS after transverse loop colostomy for obstructive colorectal cancer is extremely rare. According to PubMed search engine, we found the literature of obstructive colorectal cancer with ALS has never been reported and identified. A few cases of gastric cancer with ALS after gastrectomy with Billroth‐II or Roux‐en Y reconstruction.^4^, ^5^

T‐colostomy in this case caused traction of afferent loop at the level of Treitz ligament, followed with angulation and partial obstruction of afferent loop. ALS may lead impairment of bile and pancreatic drainage while the pressure in afferent loop raised up to 18 cm H2O.^6^, ^7^ The abnormal laboratory data such as elevated WBC, amylase and total bilirubin were compatible with pathologic change.^8^, ^9^ CT scan is diagnostic tool for ALS, especially to define the obstruction level.^10^, ^11^ Managements of acute afferent syndrome can be surgical or nonsurgical. Nonsurgical procedures include external drainage or internal drainage. Internal drainage is done with endoscopic stent in stenotic lesion.^12^, ^13^ External drainage is done with percutaneous bowel drainage (PBD)^14^, ^15^ or percutaneous transhepatic duodenal drainage (PTDD)^16^, ^17^ which depends on the situations of patient.

Lee et al^16^ reported that recurrent gastric cancer with ALS had been treated with PTDD with chemotherapy. The PTDD was retained for 11 weeks. The risk of ascending cholangitis, fluid and electrolyte imbalance may be presented no matter the PTDD is patent or not.^18^ Nonsurgical therapy might be helpful in poor surgical risk patient or a preparation for more definite surgical intervention like this case. It was faced the dilemma of emergent surgical intervention or temporal PTDD. Patient had high the rate of postoperative complication/mortality ratio in 2 weeks just taking down and closure of the transverse stoma. To avoid the surgical contamination and other complications, PTDD should be considered. PTDD effectively managed postoperative complication and improved of the colorectal cancer patient's condition. It was still doubtful whether the afferent syndrome could be resolved. If PTDD failed, patients would go to an exploratory laparotomy, resection, and anastomosis procedure for the closure and consult with G.S. for reasons of failure including adhesion or tension in the ligament of Treitz.

Recently, hypoxic pelvic perfusion (HPP) could participate interchangeably with surgical and percutaneous approaches in unresectable recurrent rectal cancer (URRC). Tumor drug exposure in the pelvis no statistically difference between the percutaneous approach and surgical approach.^19^, ^20^ Minimally invasive techniques usually percutaneous like PTDD with target‐therapy could be effective treatment in URRC.

We reported the rare case of after transverse loop colostomy with ALS, which was successfully managed by following management of the ALS by PTDD.

## 4 CONCLUSION

Routine surgery chosen and less experience for the protective stoma is the transverse colon when we noticed the antecolic reconstruction after gastric surgery. In this case report, we found temporal PTDD seems to be effective in the treatment of benign afferent loop symptoms after T‐loop colostomy. Ileostomy instead of T‐loop colostomy may be suggested for emergent colorectal surgery in patient with previous B‐II surgery.

## AUTHOR CONTRIBUTIONS

**Tung‐Yuan Chen:** Conceptualization; data curation; methodology; writing – original draft. **Chin‐Wen Hsu:** Conceptualization; data curation; methodology. **Yee‐Phoung Chang:** Conceptualization; data curation; methodology. **Min‐Tsung Wang:** Conceptualization; data curation; methodology. **Yueh‐Jung Wu:** Conceptualization; data curation; methodology; writing – original draft. **Ching‐Hsien Wang:** Resources; visualization. **Kuan‐Yu Wang:** Resources; visualization. **Tian‐Huei Chu:** Writing – review and editing. **Yung‐Kuo Lee:** Funding acquisition; writing – original draft; writing – review and editing.

## FUNDING INFORMATION

This research was funded by Kaohsiung Armed Forces General Hospital, grant number KAFGH_D_111049; and KAFGH_D_112043.

## CONFLICT OF INTEREST STATEMENT

The authors declare that they have no conflicts of interest.

## ETHICS STATEMENT

Not applicable.

## CONSENT

Written informed consent was obtained from the patient for publication of this case report and any accompanying images.

## Data Availability

Data openly available in a public repository that issues datasets with DOIs.

## References

[1] Greenstein A . Principles and practice of surgery for the colon, rectum and anus. Gastroenterology. 1999;116:1502.

[2] Geiger TM . Gordon and nivatvongs principles and practice of surgery for the colon, rectum, and anus, 4th edition. Gastroenterology. 2020;158:1170‐1171.

[3] Kim JK , Park CH , Huh JH , et al. Endoscopic management of afferent loop syndrome after a pylorus preserving pancreatoduodenecotomy presenting with obstructive jaundice and ascending cholangitis. Clin Endosc. 2011;44:59‐64. 22741115 10.5946/ce.2011.44.1.59 PMC3363051

[4] Aoyama S , Motoori M , Miyazaki Y , et al. Successful treatment of remnant gastric cancer with afferent loop syndrome managed by percutaneous transhepatic cholangial drainage followed by elective gastrectomy: a case report. Surg Case Rep. 2021;7:219. 34585307 10.1186/s40792-021-01304-6 PMC8478987

[5] Toyokawa T , Yamashita Y , Yamamoto A , Shimizu S , Inoue T , Nishiguchi Y . Clinical evaluation of ten cases with afferent loop obstruction. Nihon Fukubu Kyukyu Igakkai Zasshi (Journal of Abdominal Emergency Medicine). 2014;34:599‐606.

[6] Yao NS , Wu CW , Tiu CM , Liu JM , Whang‐Peng J , Chen LT . Percutaneous transhepatic duodenal drainage as an alternative approach in afferent loop obstruction with secondary obstructive jaundice in recurrent gastric cancer. Cardiovasc Intervent Radiol. 1998;21:350‐353. 9688809 10.1007/s002709900277

[7] Ballas KD , Rafailidis SE , Konstantinidis HD , et al. Acute afferent loop syndrome: a true emergency. A Case Report Acta Chir Belg. 2009;109:101‐103. 19341207 10.1080/00015458.2009.11680382

[8] Desai A , Manvar A , Ho S . Afferent loop syndrome due to volvulus of the afferent limb. ACG Case Rep J. 2019;6:e00123. 31620523 10.14309/crj.0000000000000123 PMC6722374

[9] Uriu Y , Kuriyama A , Ueno A , Ikegami T . Afferent loop syndrome of 10 years' onset after gastrectomy. Asian J Surg. 2019;42:935‐937. 31301932 10.1016/j.asjsur.2019.06.008

[10] Kim HC , Han JK , Kim KW , et al. Afferent loop obstruction after gastric cancer surgery: helical CT findings. Abdom Imaging. 2003;28:624‐630. 14628863 10.1007/s00261-002-0070-y

[11] Juan YH , Yu CY , Hsu HH , et al. Using multidetector‐row CT for the diagnosis of afferent loop syndrome following gastroenterostomy reconstruction. Yonsei Med J. 2011;52:574‐580. 21623598 10.3349/ymj.2011.52.4.574 PMC3104453

[12] Kim HJ , Kim JW , Kim KH , et al. A case of afferent loop syndrome treated by endoscopic drainage procedure using nasogastric tube. Korean J Gastroenterol. 2007;49:173‐176. 18172346

[13] Kim JH . Endoscopic stent placement in the palliation of malignant biliary obstruction. Clin Endosc. 2011;44:76‐86. 22741117 10.5946/ce.2011.44.2.76 PMC3363064

[14] Yazici B , Safak AA , Pehlivan M , Gokgoz T . Case report: Percutaneous bowel drainage for jaundice due to afferent loop obstruction following pancreatoduodenectomy. Tani Girisim Radyol. 2003;9:94‐96. 14661303

[15] Kojima S , Sakai H , Yuichi G , et al. Two cases of afferent loop obstruction treated with percutaneous bowel drainage(PBD). Gan to Kagaku Ryoho. 2019;46:389‐391. 30914570

[16] Lee KD , Liu TW , Wu CW , et al. Non‐surgical treatment for afferent loop syndrome in recurrent gastric cancer complicated by peritoneal carcinomatosis: percutaneous transhepatic duodenal drainage followed by 24‐hour infusion of high‐dose fluorouracil and leucovorin. Ann Oncol. 2002;13:1151‐1155. 12176796 10.1093/annonc/mdf212

[17] Watanabe M , Kondo S , Wada T , et al. A case of percutaneous transhepatic duodenal drainage for duodenal stump suture failure after gastrectomy. Gan to Kagaku Ryoho. 2021;48:1746‐1748. 35046317

[18] Kim YH , Han JK , Lee KH , Kim TK , Kim KW , Choi BI . Palliative percutaneous tube enterostomy in afferent‐loop syndrome presenting as jaundice: clinical effectiveness. J Vasc Interv Radiol. 2002;13:845‐849. 12171989 10.1016/s1051-0443(07)61995-2

[19] Guadagni S , Fiorentini G , Clementi M , Palumbo P , Mambrini A , Masedu F . Mitomycin C hypoxic pelvic perfusion for unresectable recurrent rectal cancer: pharmacokinetic comparison of surgical and percutaneous techniques. Updates Surg. 2017;69:403‐410. 28791628 10.1007/s13304-017-0480-6 PMC5591364

[20] Guadagni S , Fiorentini G , Mambrini A , et al. Multidisciplinary palliation for unresectable recurrent rectal cancer: hypoxic pelvic perfusion with mitomycin C and oxaliplatin in patients progressing after systemic chemotherapy and radiotherapy, a retrospective cohort study. Oncotarget. 2019;10:1‐13. 10.18632/oncotarget.26972 PMC6570475 31231460

